# Computational Modeling of Gold Nanoparticle Interacting with Molecules of Pharmaceutical Interest in Water

**DOI:** 10.3390/molecules28207167

**Published:** 2023-10-19

**Authors:** Massimo Fusaro, Andrzej Leś, Elżbieta U. Stolarczyk, Krzysztof Stolarczyk

**Affiliations:** 1 Faculty of Chemistry, University of Warsaw, Pasteura 1, 02-093 Warsaw, Poland; maxitp@gmail.com (M.F.); ales@chem.uw.edu.pl (A.L.); 2 National Medicines Institute, Chełmska 30/34, 00-725 Warsaw, Poland; e.stolarczyk@nil.gov.pl

**Keywords:** gold nanoparticles, derivatives, genistein, abiraterone, ethanethiol, methanethiol, citrates, (HSAB) principle, DFT, aqueous medium

## Abstract

We derived a theory of biomolecule binding to the surface of Au*_n_* clusters and of the Au plane based on the hard soft acid base (HSAB) principle and the free electron metallic surface model. With the use of quantum mechanical calculations, the chemical potential (*μ*) and the chemical hardness (*η*) of the biomolecules are estimated. The effect of the gold is introduced via the empirical value of the gold chemical potential (−5.77 eV) as well as by using the expression (modified here) for the chemical hardness (*η*). The effect of an aqueous environment is introduced by means of the ligand molecular geometry influenced by the PCM field. This theory allows for a fast and low-cost estimation of binding biomolecules to the AuNPs surface. The predicted binding of thiolated genistein and abiraterone to the gold surface is about 20 kcal/mol. The model of the exchange reaction between these biomolecules and citrates on the Au surface corresponds well with the experimental observations for thiolated abiraterone. Moreover, using a model of the place exchange of linear mercaptohydrocarbons on 12-mercaptododecane acid methyl ester bound to the Au surface, the present results reflect the known relation between exchange energy and the size of the reagents.

## 1. Introduction

Gold nanoparticles are readily conjugated to biological molecules, e.g., protein, oligonucleotides, and antibodies, primarily due to the affinity of sulfhydryl (-SH) groups to the gold surface [1]. Gold-biomolecule conjugates have been widely incorporated into diagnostic applications, where their bright red color is used in home and point-of-care tests, such as lateral flow assays [2,3]. Gold nanoparticle coated with biomolecules can specifically target cancer cells [4]. Recently, a considerably large interest in gold particles coated with thiol compounds has been noted. Such systems are being investigated, inter alia, for drug delivery systems in living organisms [3,5]. Compounds chemically adsorbed on the surface of the gold nanoparticle core can be thought of as three-dimensional monolayer (3D SAM) systems. The great interest in nanostructures is the result of their chemical, electrical, mechanical and optical properties, which are often different and sometimes more favorable than larger structures [2]. Gold nanostructures are gold nanoparticles (a diameter of 1 to 100 nm [6]) as well as nanocrystals ranging in size from one to several hundred nanometers [6,7].

Gold nanoparticles become an intermediate structure between the microscopic size of atoms or molecules and bulk materials. In such systems, one can distinguish a nanoparticle core and a stabilizing compound layer. Modifying nanoparticles with various compounds prevent their aggregation. In the case of gold nanoparticles stabilized with alkanethiols, the stabilizing layer is the alkanethiol monolayer. A specific property of gold nanoparticles modified with an organic monolayer is their ability to accumulate a charge; therefore, they are a material useful in the catalysis of electrode processes. Gold nanoparticles are already used in catalysis, optoelectronics, chemical technology, biosensors, and the pharmaceutical industry as systems for drug delivery in living organisms. In particular, it is worth noting the ease of modification of the gold nanoparticle monolayer, which may allow one to obtain materials with the desired properties.

The S-Au bond energy of gold nanoparticles is estimated to be about 44 ± 3 kcal/mol [8] or 28 kcal/mol (or 117 kJ/mol) [9]. It was found that depending on different coverage thiol densities, the Au-S binding can vary, e.g., be stronger at higher densities and weaker at lower densities [10]. In the monolayer on a flat surface (2D SAM), each thiol occupies the same area on the cluster. The packing of a thiolated ligand in the planar Au surface is almost twice the coverage of 27 nm AuNPs [11]. This can be related to the curvature of the nanoparticle core surface, where gold atoms can interact with more than one sulfur atom of the thiol [12].

There are usually more defects in a nanoparticle monolayer than in a well-organized monolayer adsorbed on a flat (they are, e.g., steps, edges, vertices) [13]. The surface of alkanethiol monolayers on 2D and 3D SAM surfaces are better understood compared to thiol-containing drug monolayers [14].

In our recent work, we have shown that the interaction between gold-adsorbed molecules, as well as the molecule binding to the gold surface, can be predicted theoretically using both 2D SAM and 3D SAM surfaces [15,16]. The present work is a continuation of this innovative approach to research. The study focused on the modeling of new drug molecules’ interaction with gold surfaces. Drugs containing thiol groups immobilized on 2D and 3D SAM surfaces were selected for the present study. It focused on comparing the properties of the Au_*n*_–drug complex, in particular the drug–gold surface interaction.

**Figure 1:**
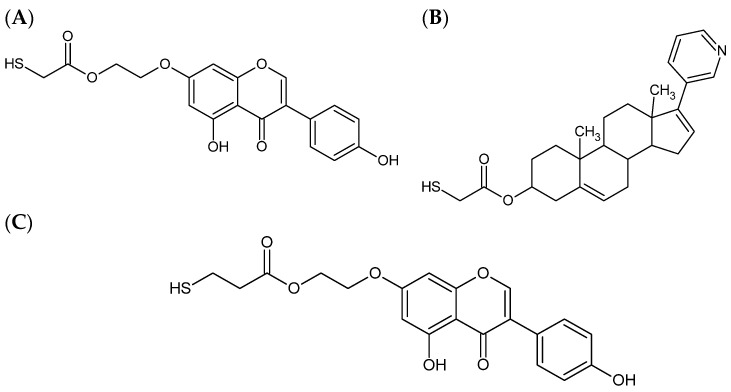
The structures of compounds using modeling research: (**A**) thiogenistein; (**B**) thioabiraterone; (**C**) ethanethiogenistein.

This paper contains the first theoretical and computational study of the recently synthesized new analog of drugs: “thiogenistein”-thiol derivative of genistein (TGE) [17] denotes the HS-CH_2_-COO-CH_2_-CH_2_-(-O)-genistein, and “thioabiraterone” denotes the HS-CH_2_-COO-abiraterone [18]. The structures are presented in Figure 1.

The present model becomes an extension of the former Gazquez et al. model [19,20], includes the water environment affecting the biomolecule-Au surface binding and includes elementary electrostatic concepts to approximate the chemical hardness (*η*) of the AuNPs. In this model, quantum mechanical calculations are only required for the prediction of the chemical potential (*μ*) and the chemical hardness (*η*) of the biomolecules. The effect of the gold enters into the theoretical equations through the empirical value of the gold chemical potential (−5.77 eV), as well as by using the expression (modified here) for the chemical hardness (*η*). The effect of the aqueous environment is introduced by means of the ligand molecular geometry influenced by the PCM field [21]. With the present modified Gazquez et al. model [19,20], it was possible to determine the upper bounds for the binding energy in the Au∷biomolecule complexes.

## 2. Results and Discussion

The present theoretical approach was designed to estimate the binding of molecules of pharmaceutical interest with the gold nanoparticles by means of fast and low-cost theoretical calculations. A goal to attain is a qualitative rather than quantitative assessment of molecule binding. It may help to answer questions from laboratory experimenters interested in having a prediction about whether there can or cannot be binding. An essential quantity of the present theory is the interaction energy between involved species. Here, the interaction energy modeling is based on the Gazquez et al. (1994) approach derived from the Density Functional Theory and the *μ*-chemical potential, *η*-hardness, (1/*η*)-softness concepts and *f*_Ai_-condensed Fukui’s functions of the *i*-th atom in the A-molecule. The present theory incorporates the free electron metallic surface model and is also modified to include the effect of the water medium.

The *μ* and *η* parameters are obtained from the B3LYP calculations with the moderate-size basis sets and uses the three-point finite difference approximation to the first- and second derivative of the total molecular energy. The water effect is introduced implicitly via the PCM model in the course of the molecular geometry optimization.

### 2.1. The Interation Energy ΔE_int_


(1) $$\Delta E_{\mathrm{int}}=\Delta E_{\nu }+\Delta E_{\mu }$$

The term ∆*E_ν_* is the energy that corresponds to the charge transfer process between *A* and *B* species arising from the chemical potential equalization principle at a constant external potential *ν* [20].

The term ∆*E_μ_* is the energy that corresponds to a reshuffling of the charge distribution, and it is basically a manifestation of the maximum hardness principle [20] and takes place at a constant chemical potential *ν* [19].

Following Gazquez et al. [19,20], the expressions for the interaction energy in Equation (1) can be written as:

(2) $${\left(\Delta E_{\mathrm{int}}\right)}_{Ai}\approx -\frac{\frac{1}{2}\frac{f_{Ai}}{\eta_{A}}{\left(\mu_{A}-\mu_{B}\right)}^{2}+\frac{1}{2}N_{e}^{2}\eta_{B}}{f_{Ai}\frac{\eta_{B}}{\eta_{A}}+1}$$

where *N_e_* [22] can be interpreted as the square of the effective number of valence electrons participating in the interaction between *A* and *B*, while *f_i_* is the condensed Fukui’s function of the *i*-th of *A* and is equal to the derivative of the charge *q_i_* of the *i*-th atom with respect to the charge of the molecule *q*.

(3) $$f_{i}={\left(\frac{\partial q_{i}}{\partial q}\right)}_{\nu }$$

### 2.2. The Hardness of the Au_n_ Nanoparticles in the Metallic Sphere Approximation

The chemical hardness *η* is related to the capacitance *C* through the equation [23,24]:

(4) $$\eta =\frac{e^{2}}{C}$$

The factor of 1/2 in the original definition of the global hardness has been omitted here for convenience, and *e* is the electronic charge [25].

The capacitance *C* of a spherical capacitor with radius *r* is given by [25]:

(5) $$C=4\pi \varepsilon_{0}r$$

From Equations (4), it follows that the chemical hardness (*η*) is:

(6) $$\eta =\frac{e^{2}}{4\pi \varepsilon_{0}\alpha r}$$

where *α* is a geometrical correcting factor that takes into account the fact that even if, macroscopically, an AuNP surface is a sphere within a good approximation, the gold atoms are not geometrical points on a surface (e.g., the Au atomic radius is about 0.146 nm).

### 2.3. The Model of the Interaction Energy in Water

One obtains an expression that is a good approximation of the (Δ*E*)*_Ai_*:

(7) $${\left(\Delta E\right)}_{Ai}\approx -\frac{1}{2}\frac{f_{Ai}}{\eta_{A}}{\left(\mu_{A}-\mu_{B}\right)}^{2}-\frac{e^{2}}{4\pi \varepsilon_{0}\alpha r}\;(\frac{N_{e}^{2}}{2}-\frac{1}{2}{\left(\frac{f_{A_{i}}}{\eta_{A}}\right)}^{2}{\left(\mu_{A}-\mu_{B}\right)}^{2})+O(\frac{1}{r^{2}})$$

It follows that the first term in Equation (7) represents the interaction energy of the biomolecule with a gold plane and the second term represents a correction for the molecule *A* interacting through the *i*-th atom with the Au cluster of the radius *r*:

(8) $${\left(\Delta E_{\infty }\right)}_{Ai}\approx -\frac{1}{2}\frac{f_{Ai}}{\eta_{A}}{\left(\mu_{A}-\mu_{B}\right)}^{2}$$

where (Δ*E_∞_*)*_A_* is the interaction energy with a gold plane and the molecule *A*, and *μ_G_* is the chemical potential of gold (−5.77 eV). The total interaction energy Δ*E_A_* between the gold nanoparticles Au*_n_* and the molecule *A* can be obtained by replacing the condensed Fukui’s function set *f_Ai_* with a value of 1 in Equations (7) and (8); it follows that:

(9) $$\Delta E_{A}\approx \Delta E{\left(\infty \right)}_{A}-\frac{e^{2}}{4\pi \varepsilon_{0}\alpha r}\;(\frac{N_{e}^{2}}{2}+\frac{{\left(\Delta E_{\infty }\right)}_{A}}{\eta_{A}})+O(\frac{1}{r^{2}})\;\mathrm{with}\;\Delta E{\left(\infty \right)}_{A}\approx -\frac{{\left(\mu_{A}-\mu_{G}\right)}^{2}}{2\eta_{A}}$$

### 2.4. The Model of Reaction Energy in Water

We consider the following exchange chemical reaction between a molecule ligand1 bounded with the gold nanocluster Au*_n_* and a molecule ligand2:


[(Au)*_n_*—ligand1] + ligand2 → (Au)*_n_*—ligand2 + ligand1 (R1)

The reaction energy Δ*E* can be derived, in this simple model, as the difference between the interaction energy (Au)*_n_*—ligand2 and (Au)*_n_*—ligand1:

(10) $$\begin{array}{l} \Delta E=E_{(Au){}_{n}}{}_{ligand2}+E_{ligand1}-E_{(Au){}_{n}}{}_{ligand1}-E_{ligand2}+(E_{(Au){}_{n}}-E_{(Au){}_{n}})\\ =(E_{(Au){}_{n}}{}_{ligand2}-E_{ligand2}-E_{(Au){}_{n}})-(E_{(Au){}_{n}}{}_{ligand1}-E_{ligand1}-E_{(Au){}_{n}})\\ =\Delta E_{ligand2}-\Delta E_{ligand1} \end{array}$$

Equation (10) can be rewritten as:

(11) $$\Delta E\approx \Delta E{\left(\infty \right)}_{ligand2}(1-\frac{e^{2}}{\eta_{{}_{ligand2}}4\pi \varepsilon_{0}\alpha r})-\Delta E{\left(\infty \right)}_{ligand1}(1-\frac{e^{2}}{\eta_{{}_{ligand1}}4\pi \varepsilon_{0}\alpha r})=E_{{}_{ligand2}}-E_{{}_{ligand1}}$$

where:

$$E_{{}_{ligand2}}=\Delta E{\left(\infty \right)}_{ligand2}(1-\frac{e^{2}}{\eta_{{}_{ligand2}}4\pi \varepsilon_{0}\alpha r})$$

And

$$E_{ligand1}=\Delta E{\left(\infty \right)}_{{}_{ligand1}}(1-\frac{e^{2}}{\eta_{{}_{ligand1}}4\pi \varepsilon_{0}\alpha r})$$

It follows that we can make the following approximations:

(12) $$E_{{}_{ligand2}}=\Delta E{\left(\infty \right)}_{ligand2}\;\mathrm{and}\;E_{ligand1}=\Delta E{\left(\infty \right)}_{{}_{ligand1}}$$

More details on the present theory, in particular the derivation of all the expressions, can be found in the Supplementary Information.

### 2.5. Biomolecules’ Hardness and Chemical Potential in Water Medium

Since biochemical reactions occur mainly in water solution, taking into account the changes in reactivity indices, going from the gas to solvent phase permits the elucidation of the actual reactivity of the compounds within DFT for neutral and charged systems using the polarizable continuum model (PCM). The Taylor expansion of the total electronic energy *E*(Δ*N*) as a function of the number of extra electrons Δ*N* [26,27] is:

(13) $$E(\Delta N)=E_{0}+\mu \Delta N+\frac{1}{2}\eta \Delta N^{2}+O(2)$$

where, *E*_0_ is the energy of the neutral species, *μ* is the chemical potential, and *η* is the chemical hardness.

It follows that:

(14) $$E(1)=E_{0}+\mu +\frac{1}{2}\eta +O(2)$$

(15) $$E(-1)=E_{0}-\mu +\frac{1}{2}\eta +O(2)$$

Summing and subtracting Equations (14) and (15), it follows that:

(16) $$\eta \approx E(1)+E(-1)-2E_{0}$$

(17) $$\mu \approx \frac{E(1)\;-\;E\left(-1\right)}{2}$$

The calculated values, in water medium, of the chemical potential *μ* and of the chemical hardness *η* of different ligands are reported in Table 1.

### 2.6. Ligand Exchange Energies

Based on the data included in Table 1, one can predict the energy output Δ*E* of the hypothetical ligand exchange reaction (R1) according to Equations (11) and (12). The reaction energy can be simply calculated using the following Equation:

(18) $$\Delta E\approx E_{{}_{ligand2}}-E_{{}_{ligand1}}$$

the reaction (R1) is energetically favorable if, in Table 2, the row of the ligand2 is above the row of the ligand1. The energy values in Table 2 are ordered by increasing energies values.

Based on this result, one can predict that a hypothetical ligand exchange will occur when the citrates are doubly negatively charged. Such a result, coming from two independent theories, corresponds with the experimentally observed ligand exchange in the course of the formation of the AuNPs dressed with “thioabiraterone” [16].

### 2.7. Model for Small Radius of the AuNPs

The total interaction energy Δ*E*: between the gold nanoparticles Au*_n_*, the ligand can be obtained by replacing the condensed Fukui’s function *f_Ai_* with a value of 1 in Equation (2); it follows that:

(19) $$\Delta E\approx -\frac{\frac{{\left(\mu_{A}-\mu_{B}\right)}^{2}}{2\eta_{A}}+\frac{N_{e}^{2}e^{2}}{8\pi \varepsilon_{0}\alpha_{r}r}\;}{\frac{e^{2}}{4\pi \varepsilon_{0}\alpha r\eta_{A}}+1}\;$$

In order to evaluate the interaction energy Δ*E*_0_ between the gold nanoparticles Au*_n_* and the ligand for a small number n of Au*_n_* gold atoms, taking into account Equation (17), we take the limit for the radius *r* of the AuNP that tends to zero, and it follows that:

(20) $$\Delta E_{0}=\underset{r\to 0}{\lim}\left(-\frac{\frac{{\left(\mu_{A}-\mu_{B}\right)}^{2}}{2\eta_{A}}+\frac{N_{e}^{2}e^{2}}{8\pi \varepsilon_{0}\alpha_{r}r}\;}{\frac{e^{2}}{4\pi \varepsilon_{0}\alpha r\eta_{A}}+1}\;\right)=-\frac{N_{e}^{2}\eta_{A}}{2}$$

**Figure 2:**
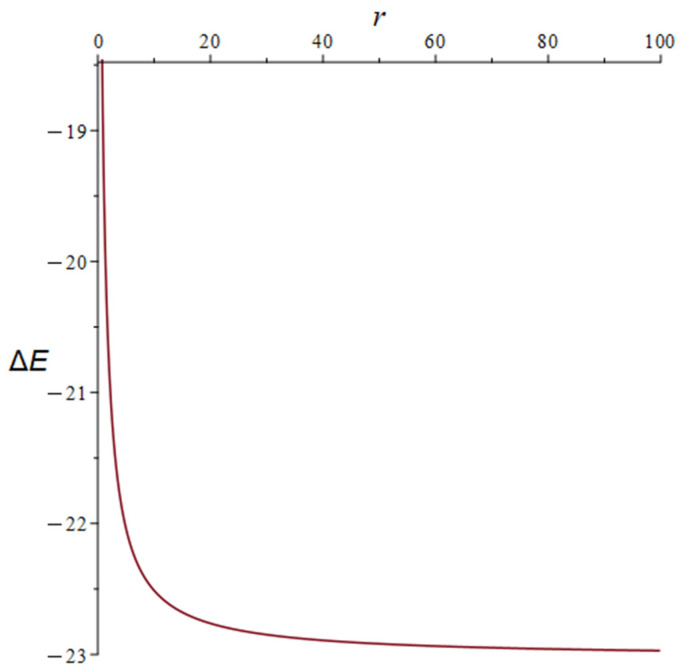
The plot of Δ*E*(*r*) in Equation (19) for the model of AuNP interacting with thioabiraterone. Δ*E* in kcal/mol, *r* in Angstroms.

The lower limit of Δ*E* in Equation (20) corresponds to *r* = 0 and becomes the right-hand side of Equation (19). The upper limit of Δ*E* corresponds to *r* = ∞ and becomes the first term in the nominator of Equation (19). The dependence of Δ*E* on *r* in Equation (19) is illustrated in Figure 2.

The calculations according to Equations (19) and (20) correspond to the case with solvent. The solvent, water in our case, implicitly enters into Equations (19) and (20) through the modification of the molecular geometry under the action of the PCM molecular field. The molecular geometry without water is somewhat different from the geometry under the PCM field; therefore, the calculated (with water) chemical potential *μ_A_* and chemical hardness *η_A_*, as well as Equations (19) and (20), lead to somewhat different results in comparison to the case when water was absent.

The theoretical (Δ*E*(*∞*))_*ligand*_ binding between the ligand and an infinite gold plane is obtained by using Equation (9).

The energy output of the reaction (R1) is favorable if Δ*E*(∞) in the row of Table 2 corresponding to the ligand2 is more negative than Δ*E*(∞) in the row with the ligand1. Consequently, the complex Au–ligand2 is energetically favorable in comparison to the complex Au–ligand1; in other words, ligand1 can be replaced by ligand2. Let us consider three examples of the Δ*E* citrate exchange on the biomolecules using the binding energies from the Table 2:

Example No. 1:

AuNP–citrate(COO^−^)_2_(-H-) + TGE → AuNP–TGE + citrate (COO^−^)_2_(--H)

Δ*E* = Δ*E*_*ligand*2_ − Δ*E*_*ligand*1_ = (−21.2) − (−18.5) = −2.7 kcal/mol;

This suggests that the ligand exchange should occur.

Example No. 2:

AuNP–citrate(COO^−^)_2_(-H-) + thioethanegenistein → AuNP–thioethanegenistein + citrate (COO^−^)_2_(--H)

Δ*E* = Δ*E*_*ligand*2_ − Δ*E*_*ligand*1_ = (−24.4) − (−18.5) = −5.9 kcal/mol;

The ligand replacement should also occur.

Example No. 3:

AuNP–citrate(COO^−^)_2_(-H-) + thioabiraterone → AuNP–thioabiraterone + citrate (COO^−^)_2_(--H)

Δ*E* = Δ*E*_*ligand*2_ − Δ*E*_*ligand*1_ = (−23.0) − (−18.5) = −4.5 kcal/mol;

The ligand replacement should also occur, as was evidenced experimentally by Stolarczyk et al. [16].

The prediction of the citrate exchange of citrate on thiogenistein or thioethanegenistein on the AuNP does not yet have an experimental counterpart in our laboratory.

It is worth noting that the citrate replacement should not occur in cases where the citrate is triply ionized, i.e., when the citrate bears the (−3) charge. These results correspond to the experimental observation of a non-complete displacement of citrate capping the AuNPs by thiolated ligands [23,29].

One can also note that in some cases (e.g., for acetate clusters) presented in Table 2, the DFT results for large Au_*n*_: acetate clusters [9] are closer to Δ*E*(*∞*) than to Δ*E*(*r*), which suggests that finite Au clusters (for a small *n*) seem to be a poor representation of the biomolecule binding to the gold surface.

The experimental estimations of the Au-S(thiol) binding of about 45 kcal/mol [24], 28 kcal/mol [9] and 30 kcal/mol for Au-S-CH_3_ [21] are not far from the estimated Au-S bond energy of about 30 kcal/mol from our models of Au:ethanethiol, Au:butanethiol and Au:octanethiol complexes (c.f. Table 2).

Another interesting observation from the data shown in Table 2 is the place exchange of the AuNP-thioalkyl conjugates, e.g., [30]. Based on the Δ*E*(*∞*) values from Table 2, one can predict that the thioalkyls with a longer CH_2_ chain can replace the thioalkyls with shorter CH_2_ chains, e.g., octanethiols (Δ*E*(*∞*) = −30.9 kcal/mol) can replace ethanethiols (Δ*E*(*∞*) = −29.4 kcal/mol) in AuNP conjugates. A similar trend is predicted for the place exchange of linear mercaptohydrocarbons on 12-mercaptododecane acid methyl ester on the Au surface. These predictions follow the trend of experimental results [30,31]. More details are provided in the Supplementary Information.

## 3. Computational Methods

We used the quantum mechanical approach using the hybrid Density Functional B3LYP method with the split valence basis set and d, p polarization functions 6-311G(d,p) (DFT:B3LYP/6-311G(d,p)) [32] for calculations of the ligands’ *μ* and *η* values.

The geometry of the models of biomolecules have been DFT: B3LYP/6-311G(d,p)-optimized. The harmonic frequencies were calculated using Gaussian 16 software [33], taking into account the solute–solvent interactions within the DFT, using the polarizable continuum model (PCM) [34] that is the default in Gaussian 16 software [33]. The water medium has been chosen due to its importance in biochemistry.

## 4. Conclusions

Although approximate in nature, the results of Au-biomolecule binding energies in water medium, derived here on the hard soft acid base (HSAB) principle and the free electron metallic surface model, are comparable to the more advanced quantum mechanical calculations for small Au_*n*_::biomolecule systems that are available. The present theory of biomolecule binding to the surface of the Au_*n*_ clusters and of the Au plane is a reasonable tool for the fast and low-cost estimation of problems that are hard to investigate experimentally. The present theory predicts a binding of about 20 kcal/mol for thiolated genistein and thiolated abiraterone with the gold surface. The predicted exchange of citrate on thiolated abiraterone occurring on the Au surface corresponds to the experimental observations. The predicted exchanges of citrate on thiogenistein and thioethanegenisten still anticipate an experimental counterpart. The predicted place exchange of linear mercaptohydrocarbons on 12-mercaptododecane acid methyl ester on the Au surface corresponds reasonably well with the known relation linking a more efficient reaction with compounds possessing a larger number of carbon atoms.

## Figures and Tables

**Table 1:** The calculated values of the chemical potential *μ_A_* and the chemical hardness *η_A_* in water medium. “thioethanegenistein” denotes the HS-CH_2_-CH_2_-COO-CH_2_-CH_2_-(-O)-genistein; “thioabiraterone” denotes the HS-CH_2_-COO-abiraterone; “thiogenistein” denotes the HS-CH_2_-COO-CH_2_-CH_2_-(-O)-genistein.

Ligand ^(1)^ A	$\mu_{A}$ eV	$\eta_{A}$ eV
octanethiol	−1.92	5.42
butanethiol	−1.91	5.43
12-mercaptododecanoic acid methylester	−2.20	4.86
ethanethiol	−1.91	5.47
methanethiol	−1.96	5.42
citrate(COO^−^)_3_	−1.56	6.95
ethanethiogenistein	−3.32	2.83
thioabiraterone	−3.10	3.57
thiogenistein	−3.41	3.03
acetate	−2.32	6.79
citrate(COO^−^)_2_ (-H-)	−2.66	6.00
citrate(COO^−^)_2_ (--H)	−2.55	6.55
citrate(COO^−^)_1_ (-HH)	−3.19	6.03
citrate(COO^−^)_1_ (H–H)	−3.25	6.50

^(1)^ “thioethanegenistein” denotes the HS-CH_2_-CH_2_-COO-CH_2_-CH_2_-(-O)-genistein; “thioabiraterone” denotes the HS-CH_2_-COO-abiraterone; “thiogenistein” denotes the HS-CH_2_-COO-CH_2_-CH_2_-(-O)-genistein; citrates code: both external carboxyl groups ionized: (-H-), one external and the central carboxyl group ionized: (--H), one external carboxyl group ionized: (-HH), the central carboxyl group ionized: (H–H).

**Table 2:** The predicted AuNP-biomolecule binding energies in water, Δ*E* (*r*), following Gazquez et al.’s theory, modified here [19,20]. Energies in kcal/mol, distances in Angstroms.

	Δ*E* (*r*) ^(1)^		
Ligand ^(2)^	Upper Limit	Closest Au–Au Distance	Comparison with DFT Calculations ^(3)^
	*r* = ∞	*r* = 2.88, in Å	
octanethiol	−31.6	−37.1	
butanethiol	−31.5	−37.2	
ethanethiol	−31.4	−37.1	
methanethiol	−30.9	−36.6	
12-mercaptododecanoic acid methylester	−30.2	−35.3	
citrate COO^3−^	−29.4	−36.8	
thioethanegenistein	−24.4	−26.9	−32.4 ^a^; −10.1 ^b^
thioabiraterone	−23.0	−27.6	−46 ^c^
thiogenistein (TGE)	−21.2	−25.1	−9.3 ^b^
acetate	−20.2	−28.8	−19.4 to −17.1 ^c,d^
citrate COO^2−^ (-H-)	−18.5	−27.0	
citrate COO^2−^ (--H)	−18.3	−27.1	
citrate COO^1−^ (-HH)	−12.8	−22.1	
citrate COO^1−^ (H–H)	−11.3	−21.1	

^(1)^ Δ*E*(*r* = ∞) (upper limit, also denoted as Δ*E*(∞)) corresponds to the binding of the AuNP(planar)-biomolecule complexes, Equation (9); Δ*E*(*r* = 2.88 Å) corresponds to the binding of AuNP-biomolecule complexes for r equal to the closest Au-Au distance in the Au(111) plane, Equation (17); The effect of water was simulated with the PCM field modifying the molecular geometry in the course of its optimization as implemented in the Gaussian 16 suite of programs. ^(2)^ “thioethanegenistein” denotes the HS-CH_2_-CH_2_-COO-CH_2_-CH_2_-(-O)-genistein; “thioabiraterone” denotes the HS-CH_2_-COO-abiraterone; “thiogenistein” denotes the HS-CH_2_-COO-CH_2_-CH_2_-(-O)-genistein; citrates code: both external carboxyl groups ionized: (-H-), one external and the central carboxyl group ionized: (--H), one external carboxyl group ionized: (-HH), the central carboxyl group ionized: (H–H). ^(3)^ The DFT calculations for the Au_25_::biomolecule complexes were performed with the DFT/6-31G(d,p) method supplemented with the lanl2dz effective potential for the Au atoms. The fixed planar geometry of Au25 cluster (5 × 5) corresponds to the fragment of the Au(111) plane with the closest Au-Au distance of *r* = 2.88 Å. The optimization of biomolecule geometry was carried out with the Berny’s algorithm implemented in the Gaussian G16 software until the geometry plateau was reached. The interaction energy was estimated by subtracting the total energy of the monomers (Au_25_, biomolecule) from the energy of the dimer (Au_25_::biomolecule). Two positions of biomolecule relative to the Au_25_ plane were studied, i.e., thiol-moiety close to the edge Au atom and almost perpendicular to the Au_25_ plane. Another DFT calculations of Al-Johani et al. [28] include acetate interacting with the 110 Au atoms forming three layers of 5 × 5, 6 × 6, and 7 × 7 atoms. ^a^ biomolecule bound to the Au_25_ plane edge; ^b^ biomolecule bound perpendicular to the Au_25_ plane; ^c^ B3LYP/6-31G(d,p) and lanl2dz for (thioabiraterone)_2_-Au_7_ estimation from ref. [16]; ^d^ model of acetate binding to Au_110_ [28].

## Data Availability

The data presented in this study are available on request from the corresponding author.

## Supplementary Materials

The following supporting information can be downloaded at: https://www.mdpi.com/article/10.3390/molecules28207167/s1, S1: Theory; S1.1: Hardness and softness; S1.2: The local HSAB principle; S1.3: Hardness of the Au_*n*_ nanoparticles in the metallic sphere approximation; S1.4: Model of the interaction energy in aqueous medium; S1.5: Model of reaction energy in aqueous medium; S1.6: Charged molecules; S1.7: Citric acid models; S1.8: Generic shape AuNP model idea.
