# Supplementary material for: Computational Modeling of Gold Nanoparticle Interacting with Molecules of Pharmaceutical Interest in Water
Source: Molecules. 2023 Oct 19;28(20):7167. doi: 10.3390/molecules28207167 (PMC10609557; doi:10.3390/molecules28207167)
Supplement: Supplementary file 1 [file molecules-28-07167-s001.zip › molecules-2619134-supplementary.pdf]

Supplementary Information.

Computational Modeling of Gold Nanoparticle Interacting with Molecules of Pharmaceutical Interest in Water.

Massimo Fusaro [1], Andrzej Leś [1], Elżbieta U. Stolarczyk [2] and Krzysztof Stolarczyk [1,*]

[1] Faculty of Chemistry, University of Warsaw, Pasteura 1, 02-093 Warsaw, Poland
[2] National Medicines Institute, Chełmska 30/34, 00-725 Warsaw, Poland
* Correspondence: kstolar@chem.uw.edu.pl

[S1] Theory

Below a derivation of the modified theory of Gazquez et al. [19,20] is presented in details.

S1.1. Hardness and softness.

Hardness, softness and Fukui function are powerful tools to predict the reactivity of a molecule. According to the density functional theory (DFT), the chemical potential μ, electronegativity χ, hardness η and softness S, of a chemical species can be represented by first and second derivatives of, E with respect to N:

$$\mu = -\chi = \left(\frac{\partial E}{\partial N}\right)_V \qquad \text{(s-1)}$$

$$\eta = \left(\frac{\partial^2 E}{\partial N^2}\right)_V = \left(\frac{\partial \mu}{\partial N}\right)_V \qquad \text{(s-2)}$$

$$S = \left(\frac{\partial N}{\partial \mu}\right)_V = \frac{1}{\eta} \qquad \text{(s-3)}$$

where E is the total electronic energy (the factor of 1/2 in the original definition of the global hardness has been omitted here for convenience) and N is the number of electrons. Considering the variation in energy in frozen MOs (molecular orbitals) using the Koopmans theorem, in a finite difference approximation we have the approximate expressions of eq. (s-1,2, 3) respectively:

$$\chi = -\mu \approx \frac{I + EA}{2} \qquad \text{(s-4)}$$

$$\eta \approx I - EA \qquad \text{(s-5)}$$

$$S \approx \frac{1}{I - EA} \qquad \text{(s-6)}$$

where I and EA are the vertical ionization energy and the electron affinity respectively.

S1.2. The local HSAB principle.

The interaction energy ΔE_{int} between two chemical species A and B with the number of electrons N_A and N_B can be written within the framework of DFT as:

$$\Delta E_{\text{int}} = E[\rho_{AB}] - E[\rho_A] - E[\rho_B] \quad \text{(s-7)}$$

where ρ_{AB}, ρ_A and ρ_B are the electron densities of the systems AB at equilibrium and of the isolated systems A and B, respectively.

It has been shown by Gazquez et al. [19] that the interaction between A and B is assumed to take place in two steps. Initially, interaction will take place through the equalization of chemical potential at constant external potential. As A and B approach the equilibrium state through changes in the electron density of the global system, changes will be generated in the external potential at constant chemical potential. This step is actually a manifestation of the principle of maximum hardness [19]:

$$\Delta E_{\text{int}} = \Delta E_v + \Delta E_\mu \quad \text{(s-8)}$$

The term ΔE_v is the energy that corresponds to the charge transfer process between A and B arising from the chemical potential equalization principle at constant external potential v [20].

The term ΔE_μ is the energy that corresponds to a reshuffling of the charge distribution and it is basically a manifestation of the maximum hardness principle [20] and takes place at constant chemical potential v [19].

Following Gazquez *et al.* [19,20], the expressions for each term in eq. s-8 can be written as:

$$\Delta E_v \approx -\frac{1}{2}\frac{(\mu_A - \mu_B)^2 S_A S_B}{S_A + S_B} \quad \text{(s-9)}$$

and

$$\Delta E_\mu \approx -\frac{1}{2}\frac{N_{AB}^2 k}{S_A + S_B} = -\frac{1}{2}\frac{\lambda}{S_A + S_B} \quad \text{(s-10)}$$

where S_A and S_B are the values of the softness of the isolated systems A and B respectively. N_{AB} is the total number of electrons of the system AB and k is the proportionality constant between S_{AB} (softness of the system AB at equilibrium) and S_A+S_B. The product of the terms N^2_{AB} and k is known as λ [19,20], and can be interpreted as the square of the effective number of valence electrons, N_e [22] participating in the interaction between A and B.

From a local point of view, if the interaction between two chemical systems A and B occurs through the i-th atom of A, one can express the interaction at the i-th atom by replacing the softness of A with the local softness of the site i in A.

The condensed local softness S_i for i-th atom is defined as:

$$S_i = S \cdot f_i \quad \text{(s-11)}$$

where f_i is the condensed Fukui's function of the i-th of A and it is equal to the derivative of the charge q_i of the i-th atom with respect to the charge of the molecule q.

$$f_i = \left(\frac{\partial q_i}{\partial q}\right)_V \tag{s-12}$$

From equations (s-8,9,10,11) it follows:

$$(\Delta E_{\text{int}})_{Ai} \approx -\frac{1}{2}\frac{(\mu_A - \mu_B)^2 S_A f_{Ai} S_B + N_e^2}{S_A f_{Ai} + S_B} \tag{s-13}$$

and from eq. (3) and eq.(13) it follows:

$$(\Delta E_{\text{int}})_{Ai} \approx -\frac{\frac{1}{2}\frac{f_{Ai}}{\eta_A}(\mu_A - \mu_B)^2 + \frac{1}{2}N_e^2\eta_B}{f_{Ai}\frac{\eta_B}{\eta_A} + 1} \tag{s-14}$$

The electrofilicity ω_a of a monovalent neutral addend *A* is defined as:

$$\omega_A = \frac{1}{2}\frac{\mu_A^{\;2}}{\eta_A} \tag{s-15}$$

It follows from eq. (14) and eq. (15):

$$(\Delta E_{\text{int}})_{Ai} \approx -\frac{\omega_A f_{Ai}(1 - \frac{\mu_B}{\mu_A})^2 + \frac{1}{2}N_e^2\eta_B}{f_{Ai}\frac{\eta_B}{\eta_A} + 1} \tag{s-16}$$

S1.3. Hardness of the Au_n nanoparticles in the metallic sphere approximation.

In order to evaluate the chemical hardness (η_B) of our gold nanoparticles Au_n models it is assumed that the gold cluster composed by *n* atoms is embedded by a sphere of radius *r*.

It follows that the radius of the sphere that is embedding *n* atoms is: $\frac{4}{3}\pi r^3 = nMm / dN_A$

$$\text{n}=\frac{4\pi}{3\cdot 16.95}r^3 = 0.2471\cdot r^3 \quad \text{and} \quad r = \sqrt[3]{\frac{3\text{n}}{4\pi}16.95} = 1.5934\sqrt[3]{\text{n}}\ \text{Å} \tag{s-17}$$

where *Mm* = 196.9665 g/mol is the gold molar mass, *d* = 19.3 g/cm^3 is the gold density and N_A is the Avogadro's number. It follows:

For n = 7 gold atoms one obtains *r* = 3.05 Å, and for *r* = 85 Å one obtains n = 151750. The capacitance *C* of a spherical capacitor of the radius *r* is given by:

$$C = 4\pi\varepsilon_0 r \tag{s-18}$$

The chemical hardness η is related to the capacitance C trough the equation [23,24]:

$$\eta = \frac{e^2}{C} \tag{s-19}$$

The factor of 1/2 in the original definition of the global hardness has been omitted here for convenience in agreement with eq. (s-2) and eq. (s-5) where *e* is the electronic charge.

From equations (19) it follows the chemical hardness (η):

$$\eta = \frac{e^2}{4\pi\varepsilon_0 \alpha r} \qquad \text{(s-20)}$$

Where α is a geometrical correcting factor that keeps into account the fact that an AuNP it is not exactly spherical.

For neutral systems, the variation in electronic chemical potential induced by solvation will approximately vanish.

According with our calculations this is true if the geometry of the molecules is kept fixed and it is not optimized in the solvent.

We DFT/Lanl2dz optimized the geometry of an Au_{60} nanoball, the resulting optimized geometry radius was 6.81 Å. We made than a single point calculation PCM in water. The geometrical correcting factor α in water was estimated to be 4.2.

S1.4. Model of the interaction energy in aqueous medium.

In order to evaluate the interaction energy $(\Delta E)_{Ai}$: between the gold nanoparticles Au_n and the molecule *A* interacting through the *i*-th atom, the condensed Fukui function f_{Ai} for the *i*-th atom of the molecule *A* has been used (maximum value of f_{Ai} is 1). From the eq. (s-16) and eq. (s-20), keeping in account that the chemical potential μ_B of our models of Au_n nanoparticles is the chemical potential of gold eq. (s-16) we replace it with μ_G and one obtains the eq. (s-21):

$$(\Delta E)_{Ai} \approx -\frac{\omega_A f_{A_i} (1 - \frac{\mu_G}{\mu_A})^2 + \frac{N_e^2 e^2}{8\pi\varepsilon_0 \alpha r}}{\frac{f_{A_i}}{\eta_A} \frac{e^2}{4\pi\varepsilon_0 \alpha r} + 1} \qquad \text{(s-21)}$$

In a simple free electron model of the gold nanoparticles, the chemical potential is the Fermi energy at zero Kelvin and it is independent from the size of the gold nanoparticles because it depends only from the density of free electrons in the metal. The previous formula (s-21), shows that, in our simplified model, the interaction energy with gold nanoparticles is independent from the fact that the spherical gold nanoparticle is hollow or full of gold atoms. Keeping in account that the maximum value of the condensed Fukui function f_{Ai} for the *i*-th atom of the molecule *A* is $f_{Ai} = 1$ as it can be deduced from its definition eq. (s-12) and the minimal value of η_A for molecules considered in the present work, i.e. $\eta_{Amin}=2.83eV$ for ethanethiogenistein, Table 1, and that the minimum value of the AuNPs radius *r* can be set to r_{min}=1 nm [6] and also that α=4.2 it follows that the denominator of eq. (s-21) can be approximated with the unity. Moreover, the maximal value (max) of the first term in the denominator of eq (s-21) can be as follows:

$$\max\left(\frac{f_{A_i}}{\eta_A} \frac{e^2}{4\pi\varepsilon_0 \alpha r}\right) = \frac{1}{\eta_{A_{\min}}} \frac{e^2}{4\pi\varepsilon_0 \alpha r_{\min}} \qquad \text{(s-22)}$$

The numerical value of the expression in eq. (s-22) is negligible comparing to the unity:

$$\frac{1}{\eta_{A_{\min}}} \frac{e^2}{4\pi\varepsilon_0 \alpha r_{\min}} = 0.12 < 1 \qquad \text{(s-23)}$$

Such a property allows expand asymptotically expression in the eq. (s-21) vs. 1/r. One obtains the expression being a good approximation to the $(\Delta E)_{Ai}$:

$$(\Delta E)_{Ai} \approx -\omega_A f_{A_i}(1-\frac{\mu_G}{\mu_A})^2 - \frac{e^2}{4\pi\varepsilon_0 \alpha r}(\frac{N_e^2}{2} - \frac{\omega_A f_{A_i}^2(1-\frac{\mu_G}{\mu_A})^2}{\eta_A}) + O(\frac{1}{r^2}) \qquad \text{(s-24)}$$

It follows that the first term in eq. (s-24) represents the interaction energy of biomolecule with a gold plane (r = ∞) and second term represents a correction for the molecule A interacting through the i-th atom with the Au cluster of the radius r. The first term reads as follows , eq. (s-25):

$$(\Delta E_\infty)_{Ai} \approx -\omega_A f_{A_i}(1-\frac{\mu_G}{\mu_A})^2 \qquad \text{(s-25)}$$

where μ_G is the chemical potential of gold (-5.77 eV), it is indirectly obtained through eq. (s-4) from the data of the ionization energy and the electron affinity of gold atom reported in the quoted paper.

The total interaction energy ΔE_A between the gold nanoparticles Au_n and the molecule A can be obtained replacing in eq. (s-24) and eq. (s-25) the condensed Fukui's function set f_{Ai} = 1 that is equivalent to replace in eq. (s-11) the condensed local softness with the softness, it follows:

$$\Delta E_A \approx (\Delta E_\infty)_A - \frac{e^2}{4\pi\varepsilon_0 \alpha r}(\frac{N_e^2}{2} + \frac{(\Delta E_\infty)_A}{\eta_A}) + O(\frac{1}{r^2}) \qquad \text{(s-26)}$$

with:

$$(\Delta E_\infty)_A \approx -\omega_A(1-\frac{\mu_G}{\mu_A})^2 = -\frac{(\mu_A-\mu_G)^2}{2\eta_A}$$

where $(\Delta E_\infty)_A$ is the interaction energy with a gold plane and the molecule A.

S1.5. Model of reaction energy in aqueous medium.

We consider the following exchange chemical reaction between a molecule ligand1 bounded with the gold nanocluster Au_n and a molecule ligand2:

[(Au)n – ligand1] + ligand2 → (Au)n –ligand2 + ligand1 (R1)

The reaction energy ΔE can be derived, in this simple model, as the difference of the interaction energy between (Au)n –ligand2 and (Au)n –ligand1:

$$\begin{aligned}\Delta E &= E_{(Au)_n ligand2} + E_{ligand1} - E_{(Au)_n ligand1} - E_{ligand2} + (E_{(Au)_n} - E_{(Au)_n}) = \\ &= (E_{(Au)_n ligand2} - E_{ligand2} - E_{(Au)_n}) - (E_{(Au)_n ligand1} - E_{ligand1} - E_{(Au)_n}) = \\ &= \Delta E_{ligand2} - \Delta E_{ligand1}\end{aligned} \qquad \text{(s-27)}$$

keeping in account that ΔE_{ligand} is the formation energy for the bond between B and the Au_n gold nanocluster schematized by the reaction:

$(Au)_n$ + ligand → $(Au)_n$ –ligand (R2)

Assuming that the effective number of valence electrons [15] participating in the interaction Ne it depends mainly on the size of the gold nanocluster, we can make the assumption that Ne is as a constant in the reaction (R1) (the radius r of the AuNP does not change in the reaction), it follows from eq. (s-26,27):

$$\Delta E \approx \Delta E_{ligand2} - \Delta E_{ligand1} = (\Delta E_{\infty})_{ligand2} - (\Delta E_{\infty})_{ligand1} - \frac{e^2}{4\pi\varepsilon_0 \alpha r}\left(\frac{(\Delta E_{\infty})_{ligand2}}{\eta_{ligand2}} - \frac{(\Delta E_{\infty})_{ligand1}}{\eta_{ligand1}}\right) \quad \text{(s-28)}$$

Equation (s-28) that can be rewritten as:

$$\Delta E \approx (\Delta E_{\infty})_{ligand2}(1 - \frac{e^2}{\eta_{ligand2} 4\pi\varepsilon_0 \alpha r}) - (\Delta E_{\infty})_{ligand1}(1 - \frac{e^2}{\eta_{ligand1} 4\pi\varepsilon_0 \alpha r}) = E_{ligand2} - E_{ligand1} \quad \text{(s-29)}$$

where:

$$E_{ligand2} = (\Delta E_{\infty})_{ligand2}(1 - \frac{e^2}{\eta_{ligand2} 4\pi\varepsilon_0 \alpha r})$$

and

$$E_{ligand1} = (\Delta E_{\infty})_{ligand1}(1 - \frac{e^2}{\eta_{ligand1} 4\pi\varepsilon_0 \alpha r}) \quad \text{(s-30)}$$

From eq. (s-29) it follows that reaction (R1) is energetically favorite if the reaction energy ΔE is negative i.e.

if :

$$E_{ligand1} > E_{ligand2} \quad \text{(s-31)}$$

Keeping in account eqs. (s-22) and eq. (s-23) it follows that the minimum value (min) of the terms in parenthesis in eq. (s-30) is:

$$\min\left(1 - \frac{e^2}{\eta_{ligand} 4\pi\varepsilon_0 \alpha r}\right) = 1 - \max\left(\frac{e^2}{\eta_{ligand} 4\pi\varepsilon_0 \alpha r}\right) = 1 - \left(\frac{e^2}{\eta_{\min} 4\pi\varepsilon_0 \alpha r_{\min}}\right) 1 - 0.12 = 0.88 \quad \text{(s-32)}$$

It follows we can make the approximations

$$E_{ligand2} = (\Delta E_{\infty})_{ligand2} \quad \text{and} \quad E_{ligand1} = (\Delta E_{\infty})_{ligand1} \quad \text{(s-33)}$$

An illustration of the ligand exchange reaction based on the Eq. (R1) was presented in the main body of the manuscript.

There is also another interesting reaction, related to the place exchange [30,31]. Let consider two more reactions, i.e. (R3) and (R4):

[(AuNP – ester] + C_nS → [AuNP – C_nS] + ester (R3)

where n = 1, 2, 4, 8 are thioalkyls with $(CH_2)_n$ groups and the ester in [(AuNP – ester] denotes mercaptododecanoic acid methylester, as ligand1.

The C_nS in [AuNP – C_nS] denote methanethiol, ethanethiol, butanethiol and octanethiol, as ligand2. According to the eq. (s-27) one can estimate the energy output of the reaction (R3) with the use of the ΔE_{∞} values presented in the Table 2.

The energy output of the reactions in the eq. (R2) is negative ($\Delta E < 0$) which suggests the prevalence of the right-hand side of the reaction (R3).

A similar reaction can be considered where in place of [(AuNP – ester] we substitute the C_nS with various values of n :

$$[(AuNP - C_nS] + C_kS \rightarrow [AuNP - C_kS] + C_nS] \qquad (R4)$$

where $k > n$. For example, for n = 2 and k = 3, 4, 8 it is predicted that in the AuNP dressed with ethanethiol. Its ligand will be replaced by larger size propanethiol, butanethiol or octanethiol.

S1.6. Charged molecules.

The total electronic energy as a function of the number of extra electrons ΔN=+-1 has been calculated, for the optimized geometry for our models of biomolecules (at ΔN = 0), using the Gaussian 16 software with the PCM option using water as medium.

S1.7. Citric acid models.

For our calculations we have used different models of citric acid keeping in account the properties of the three carboxylic groups in water solution. A short notation has been introduced to distinguish between the isomer forms. If we want e.g. to distinguish between the two citrate $(COO^-)_2$ isomers i.e. a citric acid molecule whose carboxylic groups have lost two protons we indicate one of the two isomers as (-H-) meaning that the negative charges are on the two equivalent carboxylic groups on the opposite sides of the molecule.

S1.8. Generic shape AuNP model idea.

We are aware of the shape of the gold nanoparticles which can be in some cases rather far from a ball-shape as used here. The present theory can be modified along the lines shown below, although a more complete theory is under development.

One can follow a basic equations of electroststatics. In particular, it was shown that the capacitance C of a conducting body of maximum radius R following the inequality:

$$C \le 4\pi\varepsilon_0 R \qquad (s\text{-}34)$$

It follows from equations (s-19):

$$\eta \ge \frac{e^2}{4\pi\varepsilon_0 R} \qquad (s\text{-}35)$$

where η is the chemical hardness for all the AuNPs of any shape and maximum radius R.

In equation (s-22,23) we were evaluating the validity of the asymptottic expansion of equation (s-21).

In the of a case of an AuNPs with a generic shape, equation (s-23) has to be replaced using equation (s-36) with:

$$\frac{1}{\eta_A}\frac{e^2}{4\pi\varepsilon_0 R} \ll 1 \qquad (s\text{-}36)$$

Where η_A is the chemical hardness of the molecule A interacting with the AuNP of maximum radius R.

If the inequality (s-36) is satisfied it is possible to approximate the interaction energy with the first term of the asymptotic expansion of the equation (s-21), i.e. with the planar plane approximation of the interaction energy expressed by equation (s-25).
